# Supplementary material for: Targeting a malaria merozoite surface protein with mRNA vaccine generates multifunctional antibodies
Source: Front Immunol. 2026 Sep 16;17:1840217. doi: 10.3389/fimmu.2026.1840217 (PMC13624079; doi:10.3389/fimmu.2026.1840217)
Supplement: Supplementary file 1 [file DataSheet1.docx]

**Targeting a malaria merozoite surface protein with mRNA vaccine generates multifunctional antibodies**

Thomas A. et al, 2026

**Supplementary material**

**Figure S1. *Pf*MSP2 (3D7) mRNA vaccine longevity**

ELISA analysis of total IgG decay post-immunisation in total mouse sera at 1/12800 dilution (n=5). Error bars represent median ± IQR and assays performed in duplicates.

**Figure S2. *Pf*MSP2 FC27 mRNA expression validation**

Western blot of time-course total Expi293F cell lysate expressing *Pf*MSP2 FC27. The NC membrane was probed with mouse α-Myc monoclonal antibody (Cell Signaling; 1/1000 dilution).

**Table S1.** mRNA-LNP characterisation

| **mRNA-LNP**  **formulation** | **Particle Size**  **(z-average, nm)** | **Polydispersity index (PDI)** | **Encapsulation Efficiency (EE%)** |
| --- | --- | --- | --- |
| *Pf*MSP2 3D7 | 87.983 | 0.149 | 92.193 |
| *Pf*MSP2 FC27 | 88.553 | 0.111 | 94.233 |
